# Editorial: The Management of Emotions in Sports Organizations

**DOI:** 10.3389/fpsyg.2020.607041

**Published:** 2020-10-27

**Authors:** Manuel Alonso Dos Santos, Ferran Calabuig Moreno, Irena Valantine

**Affiliations:** ^1^Marketing and Market Research Department, University of Granada, Granada, Spain; ^2^Administration Department, Universidad Católica de la Santísima Concepción, Concepción, Chile; ^3^Physical Education and Sports Department, University of Valencia, Valencia, Spain; ^4^Department of Sports Management, Economics and Sociology, Lithuanian Sports University, Kaunas, Lithuania

**Keywords:** sports organization, sports management, emotion, sports economy, sports marketing

Emotions are a relevant factor in consumer behavior (Calabuig Moreno et al., 2015; Foroughi et al., 2016). The context of sport consumption is especially suitable for the study of the effect of emotions on different variables relevant to sport management (Biscaia et al., 2012). However, there are still few empirical studies that analyze the consumption of emotions in sport. This special issue presents a compendium of articles on management, marketing, and economics of emotion sport with the aim of advancing in the application of new frontier knowledge, tools, and methods in the area of sports management.

This special issue consists of 12 articles. The first manuscript of this section is entitled “*Bullying Trends inside Sport: when Organized Sport does not Attract but Intimidates*.” The objective of Vveinhardt and Fominiene was to examine the causes of the development of intimidation in and around sporting organizations. This research revealed three levels of determinants that favor bullying: the micro level—interrelationships; the mezzo level—sports professionals' (coaches') behavior; and the macro level—management of interrelationships. The authors recommend actions leading to the recognition of bullying inside the organization and improving the competencies of the management of interrelationships in order to protect the victims of bullying and avoid new cases.

The second manuscript is titled: *Emotions and Sport Management: A Bibliometric Overview*. The authors use a bibliometric performance analysis and graphic mapping to provide a general vision of the academic research into emotions in the field of sports management. The results show that the U.S.A is the country that contributes most authors in the field and that it is a field still regarded as incipient (Baier-Fuentes et al.).

Mastromartino and Zhang published the manuscript titled *Affective Outcomes of Membership in a Sport Fan Community*. The objective was to carry out a review of the state of the research in the field of emotions and the sense of belonging to a sport fan community. The authors underline the need to monitor factors at both a technological and group or social level due to the dynamism of the communications and technology sector in order to detect changes in the sense of belonging.

The fourth manuscript is titled *Sport Spectatorship and Health Benefits: A Case of a Japanese Professional Golf Tournament*.  Watanabe et al. measure how the factors of core product (player attraction, event attractiveness, and course characteristics) and peripheral spectator services (event services, event information, event amenity, and parking and transportation) influence spectators' length of stay at a golf event, physical activity and life satisfaction. The tournament services influence physical activity, that could finally influence life satisfaction. As a consequence, the authors recommend incorporating health and well-being initiatives in the commercialization plans of sporting entities.

Tur-Porcar and Ribeiro-Soriano examine the criteria that are most affected by the commitment of athletes to sport organizations in the paper titled *The Role of Emotions and Motivations in Sport Organizations*. The authors employ the Analytic Hierarchy Process method and conclude that emotion and motivation are the most important factors in generating commitment within sport organizations. The authors recommend increasing the social motivation of the clients through improvements in well-being accompanied with team results and competitive victories, and improving emotional management through self-evaluation.

The sixth paper is titled *Portrait of Boredom Among Athletes and Its Implications in Sports Management: A Multi-Method Approach*. Velasco and Jorda present two studies with the aim of examining which aspects and incidents of being an athlete lead to boredom and how this negative emotion influences performance, attitudes and behavior. The first study reveals that boredom is prevalent amongst athletes, especially produced by carrying out repetitive tasks, lack of motivation and anticipated negative mood. The second study showed that boredom negatively influences performance and influences the over-consumption and alteration of sporting diets. Thus, the authors recommend that coaches search for signs of boredom through his or her sportspeople's consumption and the use of new technologies or materials to influence novelty and reduce boredom, as well as developing social relationships inside the group.

Kaplánová published the manuscript titled *Financial Awards and Their Effect on Football Players' Anxiety and Coping Skills* which examines the effect of economic rewards on the anxiety, coping skills and stress of players. The results of the experiment showed that the players subjected to economic rewards developed greater motivation, better physical and mental preparation and a greater inclination to respect the decisions of the coach. However, the players subjected to rewards suffer greater cognitive anxiety, finally affecting their sporting performance.

Shakina et al. presented the paper *Football Fans' Emotions: Uncertainty Against Brand Perception*. This paper examines the influence of the emotion of competition in a football match and the place where it is held on the attendance, ticket price and emotional state. Having analyzed more than 1,100 football matches from three seasons of the Brazilian state championship, the authors conclude that the price of the tickets does not have a significant influence on the attendance in a moment when the team playing is popular, thus demonstrating the emotional influence over the economic in the decision system of the spectator. Furthermore, the results showed that attendance does not vary according to the competition of the match; the attraction of the team itself on the fan is stronger than the attraction of the competition during the match.

The ninth paper is titled *Overall Quality of Sporting Events and Emotions as Predictors of Future Intentions of Duathlon Participants* and analyses the moderating effect of emotion on the influence of quality, satisfaction, and perceived value on intention to attend a sporting event. The authors apply a QCA and SEM and reach the conclusion that quality and satisfaction are key factors in predicting intention to attend. The combination of these factors with emotion explain, in themselves, the intention to attend (Magaz-González et al.).

The tenth article is titled *The Influence of Emotion in the Management of Amateur Football Organizations*. This paper examines the influence of organizational variables on credibility, identification and loyalty to amateur sporting organizations. The results showed that the organizational variables explained behavior better than the emotional variables, but that their inclusion improves prediction (Hebles et al.).

The paper titled *Future intentions of fitness center customers: effect of emotions, perceived well-being and management variables* is the eleventh. García-Pascual et al. measure the influence of management variables (i.e., quality, satisfaction and perceived value), demographic variables (i.e., age and gender) and psychological variables (i.e., personal wellbeing and emotion) on future behavior of fitness center consumers. The authors conclude that the management variables predict future behavior better, to the detriment of the psychological ones. The authors recommend frequently monitoring levels of satisfaction and quality and implementing programs that emphasize positive emotions of employees and clients.

The last paper is titled *Social Atmospherics, Affective Response, and Behavioral Intention Associated With Esports Events* and aims to examine the relationship between the social atmosphere, affective response and the intention to attend a sporting event. As a result, Jang et al. conclude that cheering behavior, similarity, cosplay, and social density (social atmospherics) influenced affective responses and behavioral intention. The authors recommend sporting managers consider atmosphere as a trigger of affective response and include animation and cosplay promotions.

The editors of this special issue would like to thank all the authors for their contributions and recognize all the future research that still needs to be done, for example through the application of neurophysiological techniques that allow emotions to be more validly recorded, research into the drivers that open up new sport markets, or new ways of social communication based on communication technologies.

## Author Contributions

All authors listed have made a substantial, direct and intellectual contribution to the work, and approved it for publication.

## Conflict of Interest

The authors declare that the research was conducted in the absence of any commercial or financial relationships that could be construed as a potential conflict of interest.
